# Ultrasound, electromyography, and balloon guidance for injecting botulinum toxin for cricopharyngeal achalasia: A case report

**DOI:** 10.1097/MD.0000000000024909

**Published:** 2021-03-19

**Authors:** Jian-Min Chen, Yang-Jia Chen, Jun Ni, Zhi-Yong Wang

**Affiliations:** Department of Rehabilitation Medicine, The First Affiliated Hospital of Fujian Medical University, 20 Chazhong Road, Fuzhou, China.

**Keywords:** achalasia, botulinum toxin, cricopharyngeal muscle, dysphagia, injection

## Abstract

**Introduction:**

Botulinum toxin (BTX) injection is a widely used treatment option for dysphagia associated with cricopharyngeal (CP) muscle achalasia, but uniform standards and protocols for administration techniques and injection sites are still lacking. This case study suggests that a unique administration technique involving a combination of ultrasound, electromyography, and balloon guidance for injecting the CP muscle can reduce inadvertent migration of BTX to non-injected tissues and increase the effectiveness and safety of BTX treatment.

**Patient concerns:**

We describe the case of a 74-year-old man who could not swallow food or saliva for 8 months.

**Diagnosis:**

The patient showed signs of true bulbar paralysis, including dizziness, hoarseness, and dysphagia. The fiberoptic endoscopic evaluation of swallowing showed massive mucilage secretion and residual materials in the postcricoid region and aspiration when swallowing 1 ml of yogurt. The video fluoroscopic swallowing study showed profoundly limited epiglottic folding and CP muscle non-relaxation, despite several unsuccessful swallow attempts.

**Interventions:**

To manage insufficient relaxation opening of the CP muscle, BTX injection was performed using ultrasound, electromyography, and balloon catheter guidance. The narrow CP muscle situated above the balloon was identified as the target of injection by ultrasound.

**Outcomes:**

The patient was able to eat a soft diet. The follow-up fibrotic endoscopic swallowing study demonstrated a reduction in the amount of pharyngeal residue. The video fluoroscopic swallowing study showed that CP muscle relaxation was significantly enhanced and no penetration was shown.

**Conclusion:**

The unique administration technique with triple guidance holds several advantages, suggesting that it may be a promising treatment for CP muscle achalasia.

## 1 Introduction

The cricopharyngeal (CP) muscle is the main component of the upper esophageal sphincter^[1,2]^; it protects the airway from retrograde passage of material refluxed from the esophagus or stomach during breathing. The CP muscle can be relaxed during swallowing to allow the passage of food bolus, fluid, or gas.^[3–5]^ Numerous acute and progressive neurological conditions,^[6]^ including brainstem stroke, usually lead to dysphagia associated with CP muscle achalasia. This abnormality can increase the risk of aspiration or laryngeal penetration and impact the patient's quality of life.^[1,4]^

In addition to balloon dilatation therapy and CP myotomy, botulinum toxin (BTX) is an effective option for treatment of CP muscle spasms or dyskinesia.^[7,8]^ However, BTX treatment carries a higher rate of side effects and complications (e.g., dysphonia, worsening dysphagia).^[6,9]^ In this study, we examined a patient with dysphagia associated with CP muscle achalasia who received injection of BTX after failure to respond to conservative treatment. The patient's dysphagia was relieved successfully. The unique injection administration technique described in this report greatly improves the safety and effectiveness of BTX injection.

## 2 Case presentation

**Figure 1:**
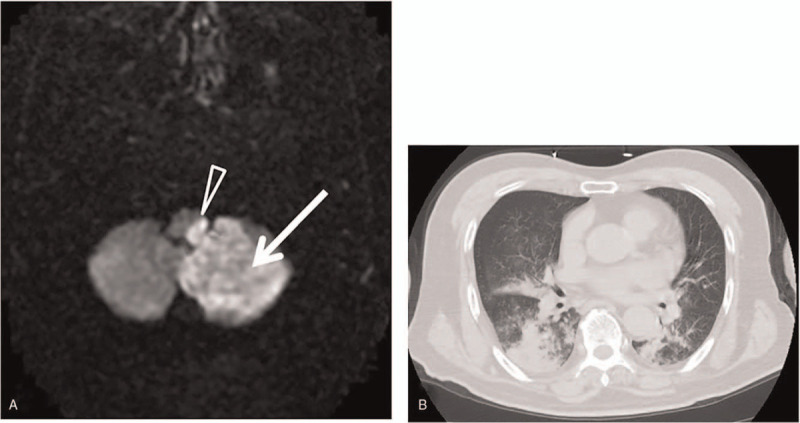
(A) MR diffusion weighted imaging suggests the acute medullary infarction in left-side cerebellar (white arrow) and medullary (white sharp triangle). (B) Sagittal plane CT of chest showed multiple inflammatory patchy shadows in both lungs.

A 74-year-old man had experienced a cerebral infarction 8 months prior. Magnetic resonance imaging of the patient's head found acute left-side cerebellar and medullary infarction (Wallenberg syndrome) (Fig. 1A). The patient showed signs of true bulbar paralysis, including dizziness, hoarseness, and dysphagia. He could not swallow saliva or food, and he was fed through a nasogastric tube. Six days after onset, the patient was transferred to the intensive care unit (ICU) for aspiration pneumonia (Fig. 1B), septic shock, and respiratory failure. He received treatment for 1 month, including a tracheotomy, assisted ventilation, and an anti-infective drug. After the tracheostomy cannula was removed, the patient was discharged in a stable condition. However, the patient was still unable to swallow saliva and frequently needed to expel foamy oral secretion, which had a serious effect on his sleep and daily life. The patient was transferred to a local rehabilitation hospital for 3 months to receive swallow training. However, he made no further improvement in swallowing and was referred to the rehabilitation department of our hospital.

**Figure 2:**
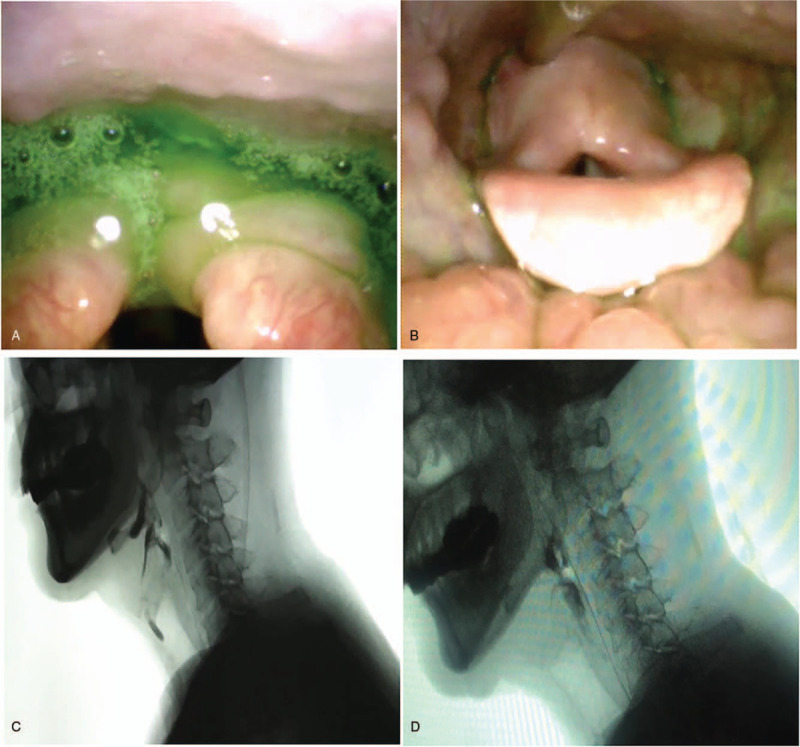
(A) Before injection, FEES revealed penetration/aspiration and pooling in the postcricoid region (red arrow). (B) After injection, FEES demonstrated the reduction in amount of pharyngeal residue. (C) Before injection, VFSS showed the case of a profoundly decreased epiglottic folding and CP muscle non-relaxation despite several invalid swallow attempts. (D) After injection, VFSS revealed a much improved CP muscle opening, decreased amount of residue, and no penetration and aspiration.

To evaluate the patient's dysphagia objectively, a fibrotic endoscopic swallowing study (FEES) and a video fluoroscopic swallowing study (VFSS) were performed. The FEES showed massive mucilage secretion and residual materials in the postcricoid region and aspiration (Fig. 2A); the fiberoptic endoscopic dysphagia severity scale (FEDSS) score was 6 (Table 1). In addition, the VFSS showed profoundly limited epiglottic folding and CP muscle non-relaxation, despite several unsuccessful swallow attempts (Fig. 2B). The functional dysphagia scale (FDS) and penetration-aspiration scale (PAS) were evaluated on the basis of VFSS video to quantitatively measure swallowing function. FDS and PAS scores were 55 and 8, respectively (Table 1). The functional oral intake scale (FOIS) was also used to evaluate the patient's swallowing function. FOIS is a 7-point scale specifically developed to reflect functional swallowing ability in stroke patients; the patient's FOIS score was 1. Consequently, the patient maintained a nasogastric tube and accepted swallow training including postural changes, compensatory maneuvers, and balloon dilatation for 1 month. However, no persistent progress in symptoms and balloon volume were observed (Table 1). By this time, the patient had experienced dysphagia for 8 months, and he complained of severe pharyngalgia due to the indwelling gastric tube and balloon expansion. He was anxious to improve his swallow function and remove the stomach tube as quickly as possible.

**Table 1:** Changes in symptoms and balloon volumes in the patient.

	FOIS score	Balloon volume	FDS score	PAS score	FEDSS score
Before injection	1	3ml	55	8	6
After injection	6	6ml	10	1	1

FDS = functional dysphagia scale, FESSS = fiberoptic endoscopic dysphagia severity scale, FOIS = functional oral intake scale, PAS = Penetration/Aspiration Scale.

**Figure 3:**
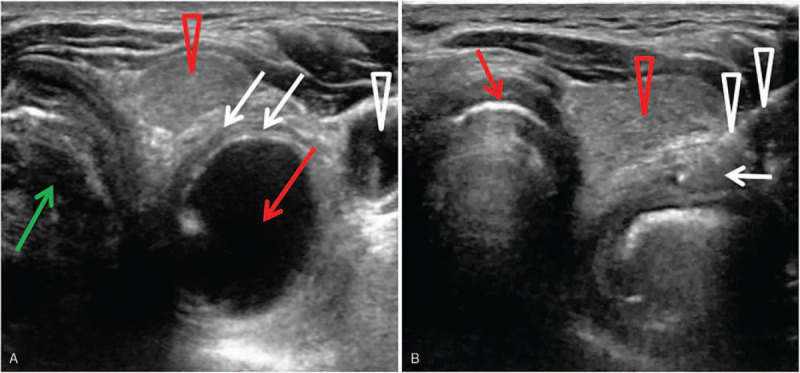
(A) An ultrasound showed that the dilated balloon of a Foley catheter was blocked and situated below the CP muscle before injection. White arrow: the CP muscle. White sharp triangle: The left common carotid artery. Red arrow: The inflated balloon below the CP muscle. Red sharp triangle: The thyroid. Green arrow: The windpipe. (B) Ultrasound-guided injection to the CP muscle and sagittal view for in-plane approach. White arrow: The CP muscle. White sharp triangle: The advancement of the needle (diameter 0.51 mm, length 60 mm). Red arrow: The thyroid cartilage. Red sharp triangle: The thyroid.

Due to insufficient swallow function progress, the patient decided to undergo a BTX injection via ultrasound, electromyography (EMG), and balloon catheter guidance to manage insufficient relaxation of the CP muscle. Written informed consent from the patient was obtained prior to BTX injection. The catheter was inserted into the esophagus through the nose, and the catheter balloon was inflated by using 5 mL of normal saline with a 10 mL syringe. The plunger was kept against the reflux of normal saline from the balloon. The catheter was gently pulled up until the balloon got stuck below the CP muscle. After the reference electrode (ipsilateral midline of the clavicle) was attached, the area between the carotid artery and the cricoid cartilage at the height of the cricoid cartilage was sterilized for the aseptic procedure. The patient lay supine with his head rotated to right, and the position of the balloon was monitored through ultrasound. The probe was initially placed axially on the patient's left neck to locate the balloon (Fig. 3A). A needle electrode cannula (monopolar electrode) was inserted via an in-plane approach into the CP muscle just above the balloon. Typical EMG signals and sounds of muscle activity were identified to obtain the accurate location of the needle. A total of 30 units of botulinum toxin type A (Botox; Allergan, Irvine, California, USA) in 0.4 mL 0.9% saline was then injected (Fig. 3B). The patient had no acute complications after the injection. After 3 days of swallowing rehabilitation therapy, the patient showed subjective improvement in ease of saliva swallowing. Seven days following BTX injection, the balloon catheter dilatation showed an increased maximum volume, and the follow-up FEES demonstrated a reduction in the amount of pharyngeal residue; the FEDSS score was 1 (Table 1). At the same time, the VFSS showed that CP muscle relaxation was significantly enhanced (Fig. 2C) and no laryngeal penetration was shown (Fig. 2D). The FDS and PAS scores were 10 and 1 respectively. As a result, the patient was able to eat a soft diet, and the FOIS score was 6. Although a small amount of residue was retained by the pyriform sinus and vallecula epiglottica, it was cleared away by multiple swallowing and compensatory maneuvers. After the injection, the patient received conventional swallowing therapy. One month after the injection, the patient's swallowing function was further improved, he could eat both solid and liquid foods. The FOIS score was 7, and the nasogastric tube was successfully removed.

## 3 Discussion

Among patients with brainstem stroke, the incidence of CP dysfunction, especially CP achalasia, is as high as 50%. Effective protocols for dysphagia management are required to avoid severe clinical complications.^[7,8]^ At present, common clinical management for CP achalasia consists of conservative approaches and surgical interventions. Swallow training, including balloon dilatation therapy, has been used relatively widely as a conservative approach in clinical settings,^[6]^ but it does not seem to be applicable for patients with long-term, severe symptoms. When conservative treatments fail, surgical management approaches such as myotomy can be effective.^[5]^ However, myotomy is highly invasive and carries risks of infection, salivary fistula formation, and recurrent laryngeal nerve injury.^[9]^ In addition, myotomy does not alter the contractile forces of the CP muscle and therefore may not benefit every patient.^[9,10]^ Since BTX injection of CP muscle was initially described in 1994,^[11]^ many studies have performed this technique. As BTX injection will primarily benefit patients with hypertonicity of the CP muscle and the ability to complete pharyngeal bolus formation, it has distinct appeal for patients who are unable or unwilling to tolerate rehabilitation training and surgical management.^[9]^ In this case report, the patient had a severe CP spasm for 8 months, which was confirmed by VFSS and FEES. Due to the patient's lack of response to rehabilitation approaches, he eventually underwent a CP BTX injection and experienced the beneficial effects of BTX.

As described by several studies, BTX is a neurotoxin that inhibits presynaptic acetylcholine release and chemically denervates the motor endplate.^[9,12]^ This results in temporary dose-related weakness or reversible palsy of the innervated muscle.^[13]^ As a result, the muscle response to chemodenervation by BTX depends on the relative myogenic (e.g., fibrosis of muscles) and neurogenic histopathologic patterns in the CP muscle. Because BTX may have a greater effect on CP muscle relaxation than neurogenic factors,^[1,9]^ it is necessary to identify abnormal tonic activity and specific alterations in electrophysiological patterns of the CP muscle with electromyographic guidance during injection.^[12,14]^

The efficacy of BTX in the management of dysphagia is likely due to several factors. Besides the CP muscle histopathology, administration technique and injection site also play important roles in determining BTX efficacy.^[12]^ However, there are no standardized techniques or protocols for administration of CP BTX.^[9]^ Use of electromyographic guidance poses a potential risk to neurovascular structures due to multiple insertions of the needle when looking for the CP muscle.^[14]^ Some studies have suggested using guidance by computed tomography scan, X-ray fluoroscopy, and endoscopy, but these methods have drawbacks: Real-time monitoring of the needle position cannot be achieved in computed tomography and X-ray fluoroscopy,^[15]^ and general anesthesia is required in endoscopy.^[16]^ Recently, the use of ultrasound has received increasing attention because it avoids the disadvantages of other guidance methods.^[4,16]^ Compared to previous studies that used ultrasound guidance,^[4,14]^ this report has two advantages. First, the injection site was determined through the triple guiding procedure of ultrasonic guidance combined with EMG and balloon catheter. The movement of CP muscle and change of EMG could be observed dynamically during the injection. Second, this study used ultrasound to identify the narrow CP muscle situated above the balloon as the target of injection. In comparison, in previous studies, the dilated balloon of the catheter was located inside the narrow CP muscle.^[16]^ CP is a C-shaped muscle and has good elastic properties^[16,17]^; muscle thickness ranges from 2.4 to 4.57 mm^[14]^ and muscle length ranges from 2 to 4 cm.^[2]^ Thus, the migration of BTX is inevitable.^[6,13]^ The change of CP muscle thickness and length during its expansion^[7]^ may lead to increased inadvertent migration of BTX to non-injected muscles, which could increase the rate of side effects and reduce the efficacy and duration of BTX effect at the intended site of action.^[13]^ Thus, the unique site of injection in this study may be a promising option for reducing the total dose of BTX, compared with many previous studies.^[9]^ In this report, only 30 units of BTX were injected into the CP muscle, and patient symptoms were relieved quickly without any side effects. However, whether administration of BTX via this technique can be generalized across patients with CP achalasia remains unclear. Future research on larger patient groups following a stroke and with other etiologies is required to confirm the benefits of this administration.

## 4 Conclusion

We report a case of dysphagia associated with CP muscle achalasia in a stroke patient. On the basis of VFSS and FEES, we suggest that neurogenic dysphagia can be treated by injection of BTX into the CP muscle under the triple guidance of ultrasound, EMG, and balloon catheter. Through this guidance program, the narrowed CP muscle can be detected, and the blood vessels and nerve branches can be visualized without using ionizing radiation during the injection. These advantages suggest that this technique may be a promising therapy for neurogenic dysphagia in patients with brainstem stroke.

## Acknowledgments

We are indebted to the subject who participated in the study for his consent and cooperation.

## Author contributions

JMC wrote the article. ZYW and YJC collected the materials. JN revised the manuscript critically for intellectual content.

**Data curation:** Yang-Jia Chen.

**Funding acquisition:** Jian-Min Chen.

**Investigation:** Zhi-Yong Wang.

**Writing – original draft:** Jian-Min Chen.

**Writing – review & editing:** Jun Ni.
